# Predictive value of various nutritional assessment scores for Short-term outcomes after emergency abdominal surgery: A prospective cohort study

**DOI:** 10.1007/s00068-025-02943-2

**Published:** 2025-08-11

**Authors:** Mohammad Hamdy Abo-Ryia, Ahmed Taher Abd Elwahab, Sherif Abel-fatah Saber, Sherif Mohamed Elgarf

**Affiliations:** Department of general surgery, faculty of medicine, Tanta University, Tanta, Egypt

**Keywords:** Nutritional assessment scores, Predictive value, Abdominal surgery, Emergency, Outcomes

## Abstract

**Background:**

Various nutritional assessment scores have been validated and accepted as predictors of postoperative outcomes in elective surgery. The objective of this study was to assess prospectively the predictive value of three of these scores (the CONUT score, the PNI, and the NRI) for short term outcomes following emergency abdominal surgery.

**Patients and methods:**

This is a prospective cohort study that was conducted over a period of 13 months. It was approved by the local ethical committee and included 150 patients who underwent emergency major abdominal surgeries and agreed to participate in the study. All patients included were clinically assessed and underwent laboratory and radiological investigations based on their presentation. Data required to calculate the three nutritional indices were collected. Patients underwent their specific surgery and received postoperative care in either the surgical ICU or surgical ward according to standard protocols based on the type of surgery.

**Results:**

Males comprised 64.70% of the study populations with a mean age of 48.48 ± 15.80 years and a mean BMI of 29.55 ± 4.99 kg/m2. The preoperative diagnosis was peritonitis due to various etiologies in 50.7% of patients, while 21.3% were diagnosed with intestinal obstruction. According to the CONUT score, the preoperative nutritional status was normal in 43.3% of patients. mild malnutrition in 46%, moderate malnutrition in 10.7%, and no patients with severe malnutrition. The PNI values ranged from 32.4 to 59.5 with a mean of 46.76 ± 6.55 while the NRI values ranged from 33.8 to 49.9 with a mean of 46.94 ± 1.90. The NRI had the highest diagnostic accuracy at 73%, followed by the CONUT score at 68%, and lastly the PNI at 66%.

**Conclusion:**

The three nutritional assessment scores were significant predictors of postoperative complications in emergency abdominal surgery, with the NRI showing the highest diagnostic accuracy.

## Introduction

Several studies have demonstrated that poor nutritional status before surgery increases the risk of postoperative morbidity, and mortality. Additionally, improving the preoperative nutritional status has been shown to improve clinical outcomes, and reduce costs in patients undergoing elective major gastrointestinal surgery [1–3]. To accurately assess nutritional status and predict postoperative complications, researchers have developed various nutritional assessment scores. These scores have been validated, and their predictive value has been evaluated through numerous retrospective studies involving various gastrointestinal malignancies [4–8]. The controlling nutritional status (CONUT) score, the prognostic nutritional index (PNI), and the nutrition risk index (NRI) are all commonly used and accepted nutritional indices for elective major abdominal surgeries [4, 6, 8]. Expansion of ERAS (Enhanced Recovery After Surgery) programs to include emergency surgery has highlighted the significance of patients’ nutritional health and its impact on surgical outcomes [9]. Most patients who need emergency laparotomy are at risk of malnutrition. Nutritional assessment is frequently overlooked and underestimated; this can lead to significant morbidity and mortality. Therefore, early identification of such patients can help to timely refer them to the dietitian resulting in improved nutritional status [10]. The aim of this study was to assess prospectively the predictive value of the CONUT score, the PNI, and the NRI for short term outcomes after emergency abdominal surgery.

## Patients and methods

This prospective observational study was conducted at the Emergency sector of the General Surgery Department at Tanta University Hospitals in Tanta, Egypt, during the period from November 2020 to December 2021. It was approved by the Institutional Ethical Committee (approval no. 34229/11/20) and included 150 eligible patients who agreed to participate and provided informed consent. The study included all adult patients who underwent emergency major abdominal surgery. Patients with ASA grade III or higher were excluded from this study because their complex medical conditions and increased risk of complications and adverse events could have hindered accurate data interpretation. Patients under 18 years old, those managed conservatively, those who underwent non-major surgical procedures (such as non-complicated appendectomy or emergency hernia repair without resection anastomosis), and those who refused to participate were excluded from the study. All patients included were clinically assessed and underwent laboratory and radiological investigations based on their presentation. Data required to calculate the three nutritional indices were collected. The CONUT score was calculated using serum albumin concentration, total peripheral lymphocyte count, and total cholesterol concentration. Based on the total scores for these three parameters, nutritional status was categorized as normal, light malnutrition, moderate malnutrition, or severe malnutrition (see Table 1) [4]. The PNI was calculated using the formula: 10 × serum albumin value (g/dl) + 0.005 × total lymphocyte count in the peripheral blood (per mm3) [6]. The NRI was calculated using the formula: NRI = (1.489 × serum albumin, g/L) + (41.7 × current weight/usual weight). Usual weight was defined as a stable weight six months or more before illness [8]. All included patients underwent their specific surgery and received postoperative care in either the surgical ICU or surgical ward. Postoperative oral intake, medications, wound care, and discharge followed standard protocols based on the type of surgery. Postoperative data, including complications and their grades according to the Clavien-Dindo [11] classification, were recorded.

**Table 1:** Assessment of the nutritional status using the CONUT score [5]

	None	Light	Moderate	Severe
Serum albumin (g/dl)	≥ 3.50	3.00-3.49	2.50–2.99	< 2.50
Score	0	2	4	6
Total lymphocyte count/mm^3^	> 1600	1200–1599	800–1199	< 800
Score	0	1	2	3
Total cholesterol (mg/dl)	≥ 180	140–179	100–139	< 100
Score	0	1	2	3
Total score	0–1	2–4	5–8	9–12

Statistical Analysis: The collected data were organized, tabulated, and statistically analyzed using SPSS^®^ statistical software for Windows. Ranges, means, and standard deviations were calculated. Statistical comparisons were made using chi-square test, t-test, or Fisher exact test, with a P value < 0.05 considered statistically significant.

## Results

**Fig. 1:**
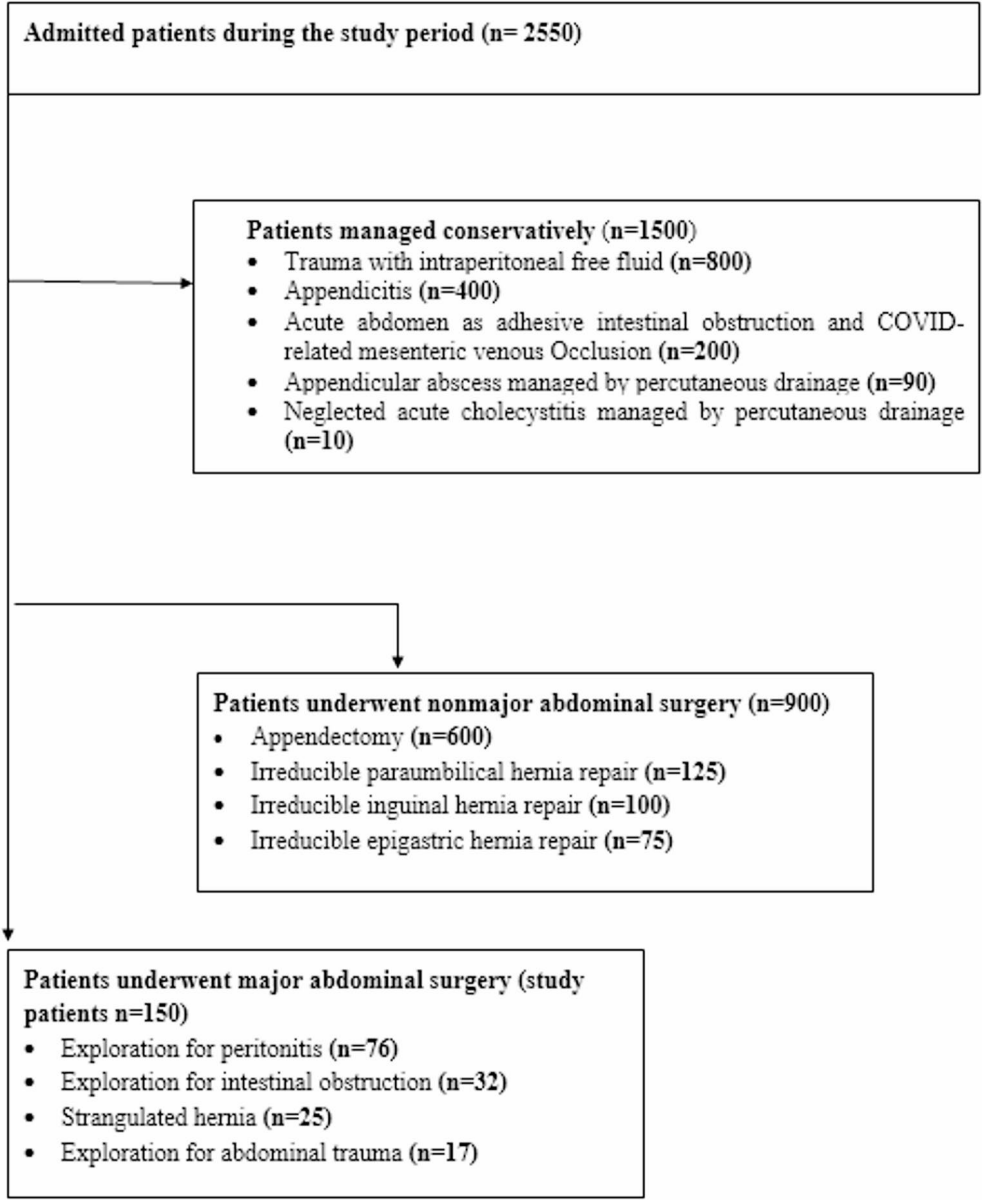
Flowchart of patient recruitment during the study

**Fig. 2:**
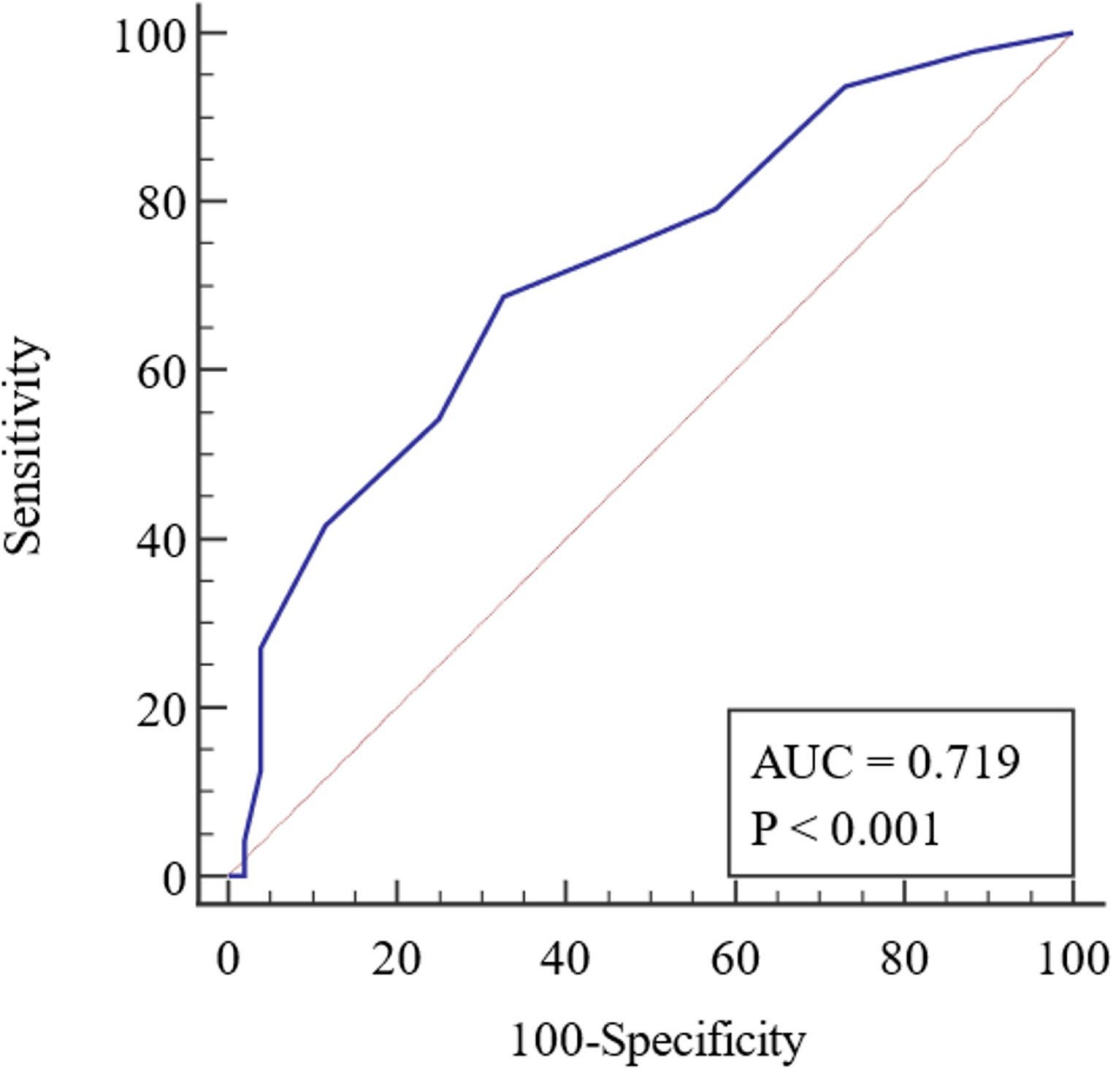
ROC curve of CONUT score to predict complications

**Fig. 3:**
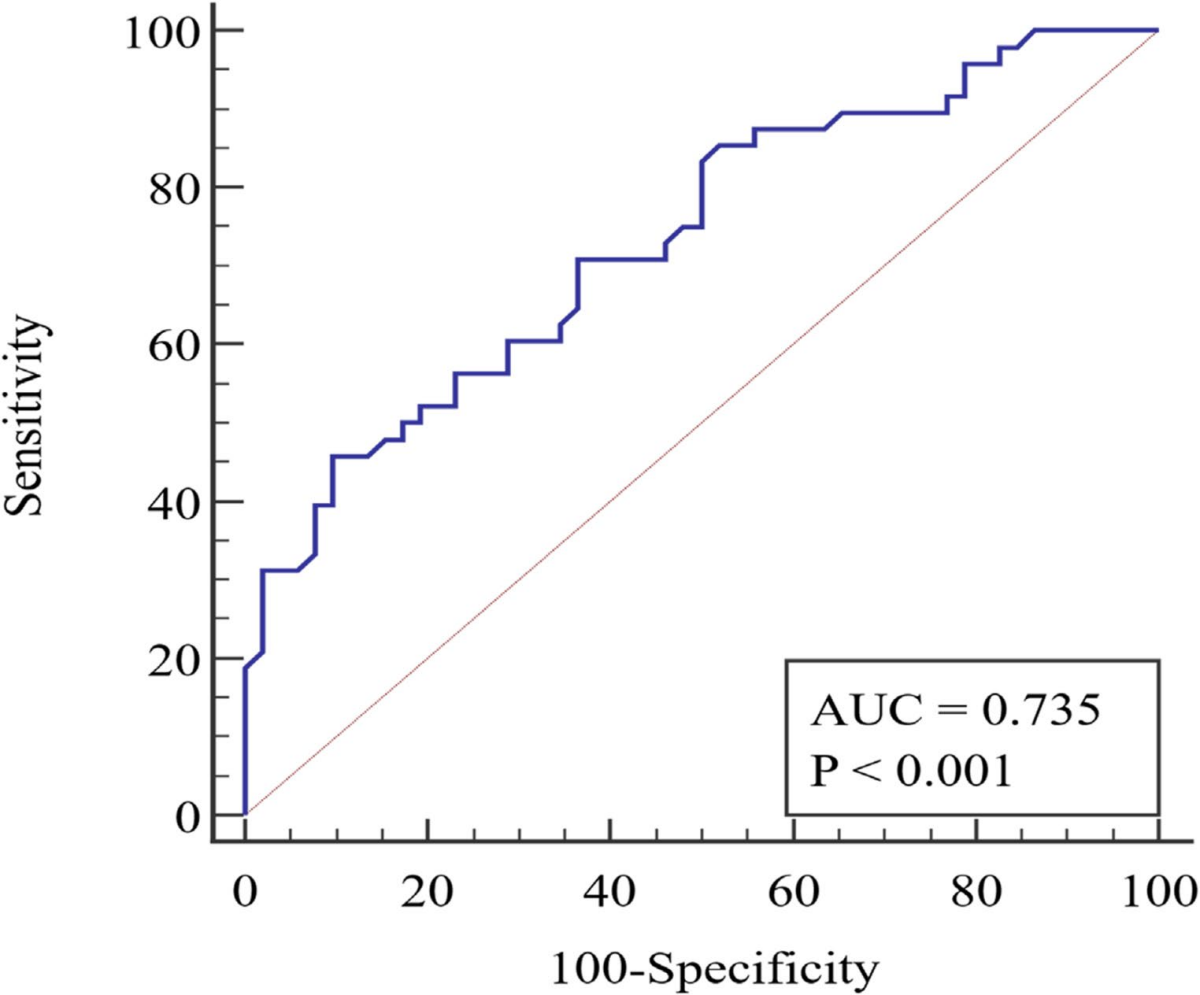
ROC curve of PNI to predict complications

**Fig. 4:**
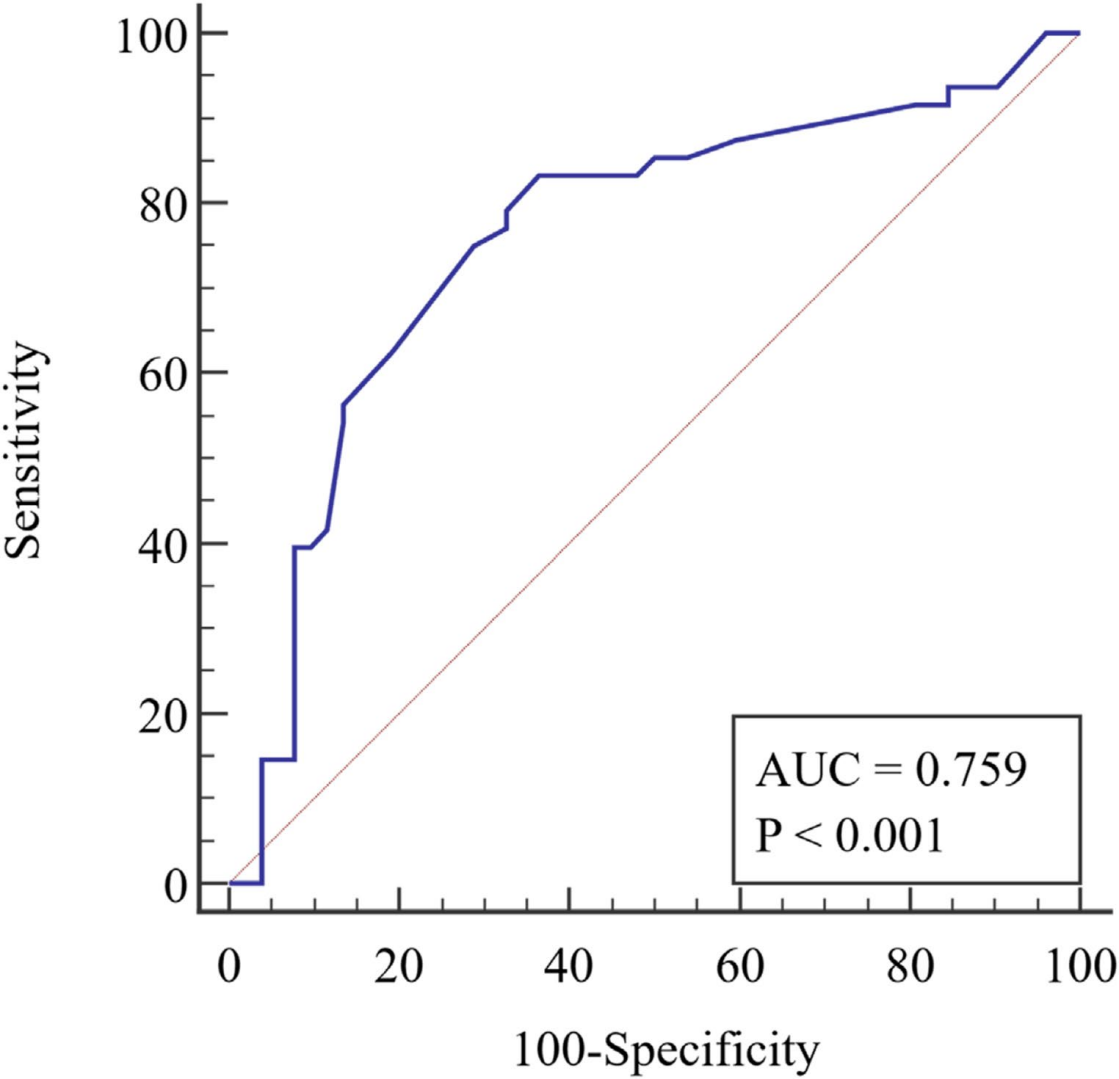
ROC curve of PNI to predict complications

During the study period, a total of 2550 emergency cases were admitted to the general surgery department’s emergency sector. Of these, 1500 cases were managed conservatively, 900 cases underwent non-major surgical intervention, and 150 patients underwent major surgical intervention and were included in the study. Figure 1 shows the flow chart of patient recruitment for this study. Of the study patients, 97 (64.70%) were male with a mean age of 48.48 ± 15.80 years and a mean BMI of 29.55 ± 4.99 kg/m2. Table 2 displays the characteristics of all patients. The mean lymphocyte count for our patients was 1961.23 ± 920.97, the mean serum albumin level was 3.72 ± 0.45 (gm/dl), and the mean serum cholesterol level was 153.69 ± 34.35 (mg/dl). Table 3 presents the patients’ laboratory data. The preoperative diagnosis for 76 (50.7%) patients was peritonitis due to various etiologies, while 32 (21.3%) patients were diagnosed with intestinal obstruction. Table 4 shows the different diagnoses and operative findings. According to the CONUT score, the preoperative nutritional status of our patients was as follows: 65 patients (43.3%) had normal nutrition, 69 patients (46%) had mild malnutrition, 16 patients (10.7%) had moderate malnutrition, and no patients had severe malnutrition. The PNI values ranged from 32.4 to 59.5 with a mean of 46.76 ± 6.55 and the NRI values ranged from 33.8 to 49.9 with a mean of 46.94 ± 1.90. Postoperative complications were reported in 113 (75.3%) patients, with surgical site infection being the most common (55.3%). Other complications included seroma (10.6%), chest infection (2%), ileus (1.3%), scrotal edema (1.3%), wound dehiscence (2%), and intra-abdominal collection (2.6%). Only one mortality (0.67%) was reported. Table 5 displays the distribution of complications according to the Clavien-Dindo classification. The hospital stays for the studied patients ranged from 3 to 15 days with a mean of 6.23 ± 1.90 days. We plotted the ROC curve for the three nutritional assessment scores (CONUT, PNI, and NRI) based on postoperative overall complications, as seen in Figs. 2 and 3, and 4. The resulting data revealed that the NRI had the highest diagnostic accuracy at 73%, followed by the CONUT score at 68%, and lastly the PNI at 66%. Full data of the three ROC curves are presented in Table 6.

**Table 2:** Characteristics of the studied patients

Variable		
Gender	Males	97 (64.7%)
	Females	53 (35.3%)
Age	Range	18–84
	Mean ± SD	48.48 ± 15.80
BMI	Range	20–44
	Mean ± SD	29.55 ± 4.99
Risk	Smoking	69 (46.0%)
	Addiction	13 (8.6%)
	Obesity	60 (40%)
Comorbidities	HTN	27 (18%)
	DM	21 (14%)
	Hepatic	9 (6%)
	Cardiac	6 (4%)
	Chronic kidney disease	6 (4%)
Surgical History	Cesarean section	18 (12.0%)
	Appendectomy	16 (10.6%)
	Ventral Hernia repair	15 (10%)
	Abdominal exploration	9 (6.0%)
	Splenectomy	2 (1.30%)
	Cholecystectomy	2 (1.30%)

BMI=body mass index, HTN=hypertension, DM=diabetes mellitus

**Table 3:** Laboratory data of the studied patients

	*N* = 150	
HB (gm./dl)	Mean ± SD	10.7 ± 1.34
	Range	7.0–14.0
Platelet (cells/dl)	Mean ± SD	264,200 ± 55,290
	Range	110,000–390,000
Albumin (gm./dl)	Mean ± SD	3.72 ± 0.45
	Range	2.7–4.9
Cholesterol (mg/dl)	Mean ± SD	153.69 ± 34.35
	Range	80–230
Total leucocytic count (cells/dl)	Mean ± SD	14,500 ± 3250
	Range	10,500–19,200
Lymphocyte (cells/dl)	Mean ± SD	1961.23 ± 920.97
	Range	104–4500
Serum creatinine (mg/dl)	Mean ± SD	1.5 ± 0.51
	Range	0.7–4.0
Serum urea (mg/dl)	Mean ± SD	34.1 ± 7.57
	Range	25.0–75.0
SGPT (U/L)	Mean ± SD	33.6 ± 9.41
	Range	25.0–70.0
SGOT (U/L)	Mean ± SD	28.5 ± 10.07
	Range	16.0–60.0

**Table 4:** Diagnosis and operative findings of the studied patients

Diagnosis	Pathology	*N* (%)
Peritonitis	Perforated peptic ulcer	36 (24.0%)
	Perforated appendicitis	24 (16.0%)
	Complicated sigmoid diverticulitis	10 (6.7%)
	Miscellaneous bowel perforation	6 (4.0%)
Intestinal obstruction	Adhesive intestinal obstruction	18 (12%)
	Colon cancer complicated with intestinal obstruction	12 (8.0%)
	Sigmoid volvulus	2 (1.3%)
Strangulated hernia	underwent resection anastomosis	25 (16.7%)
Trauma	Ruptured spleen	9 (6.0%)
	Stab abdomen with bowel perforation	6 (4.0%)
	Traumatic diaphragmatic rupture	2 (1.3%)

**Table 5:** Complications according to Clavien-Dindo classification in the patients studied

Clavien-Dindo classification	*N* (%)
I	9 (6%)
II	96 (64%)
IIIa	5 (3%)
IIIb	3 (2%)
IVa	0 (0%)
IVb	0 (0%)
V	1 (0%)

**Table 6:** Validity for the three scores to predict complications

	COUNT	PNI	NRI
AUC	0.719	0.735	0.759
Sensitivity	0.68	0.83	0.83
Specificity	0.67	0.50	0.63
PPV	66%	60.6%	67.8%
NPV	70%	76.5%	80.5%
Diagnostic accuracy	68%	66%	73%
Cut off	4	40	46.3

## Discussion

In recent decades, various nutritional assessment scores have been studied as potential predictors of postoperative complications in retrospective studies involving patients undergoing elective surgeries for various gastrointestinal malignancies. These scores have shown significance in categorizing patients based on their nutritional status and guiding interventions to improve nutrition for those with poor nutritional status, resulting in better surgical outcomes [4–7]. However, in the setting of emergency surgery, there is a lack of published research exploring this aspect [10, 12–14]. This may be due to the differences in nature between emergency surgeries and elective ones. Also, the challenges faced by seriously ill patients with neoplastic diseases who are about to undergo surgery differ from those of post-traumatic or septic patients undergoing emergency laparotomy [12]. Wide variation exists in approaches to identify malnutrition risk in emergency general surgery patients, using a range of tools and nutrition markers. The optimum screening tool to identify such patients in practice and research is yet to be determined. Raslan et al. [10] used Malnutrition Universal Screening Tool (MUST) and reported its significance in identifying patients in need of nutritional support. While Mohil et al. [12] used an old version of the prognostic nutrition index (PNI) and subjective global assessment scale (SGA) to assess nutrition and predict morbidity in emergency laparotomy patients. Akula et al. [15] used anthropometric parameters like body mass index (BMI), midarm circumference (MAC), and tissue skinfold thickness (TSFT). In addition to SGA, serum albumin, and absolute lymphocyte count (ALC).

In our hospital, preoperative nutritional assessment is a routine component of the evaluation process for elective surgeries, particularly for patients with chronic illnesses and those scheduled for major surgical interventions. These assessments are performed through our dedicated outpatient nutrition clinic. Patients’ nutritional statuses are then optimized, either on an outpatient basis or during inpatient admission, depending on their individual needs. Conversely, in emergency situations, no preoperative nutritional assessment is conducted, and a nutritionist is consulted as required postoperatively. Therefore, in this study, we evaluated the effectiveness of three commonly used nutritional indices in elective surgery to test their ability to predict outcomes in emergency abdominal surgeries. These indices were COUNT, PNI, and NRI. Our findings showed an independent association between these indices and the development of postoperative complications, with varying degrees of accuracy. This highlights the impact of nutritional status on the outcomes of emergency surgery, which has been confirmed in other studies [10, 12–14]. Out of the three indices (COUNT, PNI, and NRI), the NRI had the highest diagnostic accuracy of 73% at a cut-off value of 46.3. This was followed by the COUNT score with an accuracy of 68% at a cut-off value of 4, and the PNI with a 66% accuracy at a cut-off value of 40. It is important to note that both COUNT and PNI rely significantly on peripheral lymphocyte count, which reflects the body’s overall nutritional and immune status in elective surgeries. However, in emergency surgeries, particularly those involving sepsis, peripheral lymphocyte count is often elevated. This elevation can compromise the accuracy of COUNT and PNI, unlike the NRI, which primarily depends on weight measurements. This distinction may explain NRI’s superior diagnostic accuracy.

In this study, postoperative complications occurred in 75.3% of patients. Of these, 55.3% were surgical site infections. Other complications included seroma in 10.6% of patients, intra-abdominal collection in 2.6%, chest infection in 2%, and wound dehiscence in 2%. Byakhodi et al. [16], conducted a prospective study on post-operative morbidity and mortality in emergency abdominal surgery, which included 117 patients. They reported that 96 out of 117 patients (82.05%) experienced post-operative complications, including deaths. The most common complications were surgical site infections in 40.17% of patients, followed by pulmonary complications in 39.31%, renal complications in 29.9%, cardiac complications in 1.7%, and deep vein thrombosis in 1.7% of patients.

Research has established the value of using nutritional assessment scores in elective surgery, as it guides the correction of a patient’s nutritional status before the procedure. However, in emergency surgery, there is no opportunity to correct a patient’s nutritional status preoperatively. Nevertheless, distinguishing patients with poor nutrition is crucial to initiate immediate postoperative support, either by parenteral or enteral nutrition (EN), provided there is no contraindication to EN [13, 17, 18].

Multiple studies have suggested that early EN (EEN) is feasible and beneficial after emergency GI surgery, if there are no contraindications such as intestinal obstruction, malabsorption, multiple fistulas with high output, intestinal ischemia, severe shock with impaired splanchnic perfusion, and fulminant sepsis [17].

Key strengths of this study were its prospective methodology and the specific inclusion criteria of emergency major abdominal procedures. Additionally, the study provided a comparative analysis of three nutritional assessment tools.

However, limitations to consider are the relatively small sample size and the single-center design. Furthermore, the COUNT and PNI scores depend on peripheral lymphocyte count, which is often elevated in sepsis and thus may not accurately reflect a patient's nutritional status. Additionally, albumin, a key component of all three scores, often drops in sepsis and trauma due to increased capillary permeability, impaired liver function, and augmented inflammation.

Given these considerations, we recommend future research focusing on developing nutritional assessment scores for emergency surgery to utilize markers unaffected by either sepsis or trauma.

## Conclusion

This study concludes that the three nutritional assessment scores (COUNT, PNI, and NRI) are significant predictors of postoperative complications in emergency abdominal surgery, with the NRI showing the highest diagnostic accuracy, followed by the COUNT score and then the PNI.

## Data Availability

Data is provided within the manuscript or supplementary information files.
